# The Tripartite Interaction of Host Immunity–*Bacillus thuringiensis* Infection–Gut Microbiota

**DOI:** 10.3390/toxins12080514

**Published:** 2020-08-12

**Authors:** Shuzhong Li, Surajit De Mandal, Xiaoxia Xu, Fengliang Jin

**Affiliations:** Laboratory of Bio-Pesticide Innovation and Application of Guangdong Province, College of Agriculture, South China Agricultural University, Guangzhou 510642, China; shuzhongli@stu.scau.edu.cn (S.L.); surajit_micro@scau.edu.cn (S.D.M.); xuxiaoxia111@scau.edu.cn (X.X.)

**Keywords:** *Bacillus thuringiensis*, antimicrobial peptide, gut microbiota

## Abstract

*Bacillus thuringiensis* (Bt) is an important cosmopolitan bacterial entomopathogen, which produces various protein toxins that have been expressed in transgenic crops. The evolved molecular interaction between the insect immune system and gut microbiota is changed during the Bt infection process. The host immune response, such as the expression of induced antimicrobial peptides (AMPs), the melanization response, and the production of reactive oxygen species (ROS), varies with different doses of Bt infection. Moreover, *B. thuringiensis* infection changes the abundance and structural composition of the intestinal bacteria community. The activated immune response, together with dysbiosis of the gut microbiota, also has an important effect on Bt pathogenicity and insect resistance to Bt. In this review, we attempt to clarify this tripartite interaction of host immunity, Bt infection, and gut microbiota, especially the important role of key immune regulators and symbiotic bacteria in the Bt killing activity. Increasing the effectiveness of biocontrol agents by interfering with insect resistance and controlling symbiotic bacteria can be important steps for the successful application of microbial biopesticides.

## 1. Introduction

The Gram-positive bacterium *Bacillus thuringiensis* (Bt) and its toxins are used to control several orders of insects, including agricultural pests and pathogen vectors [1,2]. Due to their selective insecticidal activity, *B. thuringiensis* toxins have become the most widely used commercial biopesticide worldwide [3,4]. Besides, the isolated Bt toxin genes have also been expressed in several transgenic Bt crops, and these strategies have reduced reliance on chemical pesticides [5,6,7]. The most common virulence factors of Bt are the crystal (Cry) toxin proteins produced during the sporulation phase of its growth cycle when ingested by susceptible insect larvae. The Cry toxins solubilize in the gut and are further activated by the host gut protease. The active fragments cross the peritrophic membrane and bind to the protein receptor located on the brush border membrane of midgut epithelial cells and create pores that induce osmotic cell lysis and subsequent death [8,9,10]. 

The widespread use of Bt spray products in high-value horticulture and the large-scale cultivation of Bt transgenic cotton and maize has resulted in cases of field resistance in several lepidopteran pest species and the western corn rootworm, *Diabrotica virgifera virgifera* [11,12]. The most common factors associated with Bt resistance are alteration of the Bt toxin receptors’ binding site, mutations, and altered expressions of the midgut receptor genes [12,13,14]. Several Bt Cry toxin receptors, such as aminopeptidase-N (APN), alkaline phosphatase (ALP), cadherin, and ATP-binding cassette transporter (ABC transporter), have been identified and characterized in the midgut membrane of the insects [9,15,16]. *B. thuringiensis* resistance has also been linked to several other factors, such as inactivation of the midgut protease required for processing the Bt protoxins [17], gut stem cell proliferation, and differentiation [18]. However, the host immune response and the function of the gut microbiota during Bt infection, which are important aspects of Bt research, has still been inadequately studied and remains controversial [19,20,21,22]. 

The insect’s innate immune system consists of both humoral and cellular immune responses, which depend on the non-self recognition of microbes and the subsequent production of immune effectors [23]. The humoral immune response of insects includes the induction of antimicrobial peptides (AMPs), lysozymes, and the rapidly activated phenoloxidase (PO) cascade-mediated melanization [24]. AMPs are produced by two major immune pathways, Toll and IMD (immune deficiency). These pathways produce and regulate the expression of AMPs that are specific to either Gram-positive bacterial/fungal and Gram-negative bacterial infection, respectively [25,26]. The insect cellular immune process consists of encapsulation, nodulation, and phagocytosis, which is primarily driven by the hemocyte [27]. Recognition by pattern recognition receptors (PRRs) triggers immune signal transduction, and results in the activation of the Toll, Imd, Janus kinase/signal transducer and activator of transcription (JAK/STAT), c-Jun N-terminal kinase (JNK), and prophenoloxidase (PPO) pathways [28,29,30]. Furthermore, insects possess both midgut-specific and systemic immune responses to combat the infection, and reactive oxygen species (ROS) production mediated by dual oxidase (DUOX) is another inducible immune defense mechanism of insects [23]. 

**Figure 1:**
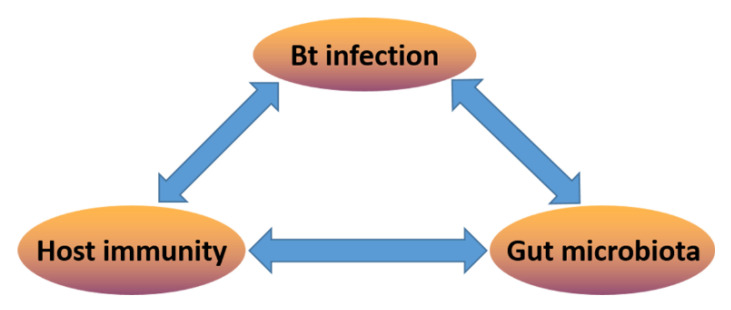
The tripartite interaction model between host immunity, Bt (*Bacillus thuringiensis*) infection, and gut microbiota.

The insect gut microbiota includes not only the bacterial community but also fungi, protists, and archaea, although the bacterial species dominate in the gut microbial community [31], and plays a vital role in insect development, nutrition, immunity, metabolism, and colonization resistance against pathogens [32,33,34,35,36,37]. Several factors, such as environmental habitat, host, developmental stage, and diet, play a significant role in the structure and function of the insect gut microbiota [38,39]. It has also been reported that Bt toxicity is often associated with the abundance of the gut microbiota. The lepidopteran pest *Spodoptera exigua* can tolerate the action of Bt toxin when it contains an increased midgut microbiota load [40]. Native gut microbiota can also stimulate the host immune system. It has been reported that the native gut microbiota of bee is associated with the upregulated expression of AMPs, such as apidaecin and hymenoptaecin [41]. This indicates that gut microbiota could help the host to maintain an appropriate immune level, and play a vital role in the survival against Bt toxicity. In this review, we describe the tripartite interaction between host immunity, Bt infection, and gut microbiota (Figure 1). We discuss the effects of Bt infection on the host immune response and intestinal microbes, and how gut microbiota responds to Bt toxicity or causes Bt resistance, as well as the mechanism used by the host to limit Bt infection while maintaining intestinal homeostasis. 

## 2. The Interaction between Host Immunity and Gut Microbiota

### 2.1. Native Gut Microbiota-Induced Host Immune Response

It is well known that the host insect immune system is stimulated upon immune challenge by invading pathogens [23]. The local gut immunity plays a vital role in maintaining gut homeostasis by inhibiting or removing invading pathogens and limiting the growth of the symbionts [42]. Native gut microbiota participates in various symbiotic interactions and also affects host immunity, but the relationship between native gut microbiota and host immune function has so far been less well studied. Kwong et al. (2007) reported that the expression of the antimicrobial peptides (AMPs) is highly upregulated in gut tissue having normal gut microbiota in comparison with the gut tissues deficient in the microbiota. They suggest that the native microbiota may induce an immune response in the host [41]. Intriguingly, colonization of one specific intestinal bacteria, *Snodgrassella alvi,* to the host alone does not induce AMPs expression. However, colonization of another gut symbiont *Frischella perrara* results in a strong host immune response involving the upregulation of AMPs and the genes associated with the melanization cascade [43]. It suggests that different microbial species may have a different regulatory function in the host. Similar to the above results, the gut commensal microbiota of Red palm weevil (RPW), *Rhynchophorus ferrugineus* Olivier can help to protect against pathogenic infection by priming the immune system [44], and the colonization of gut commensal microbiota could enhance the immunocompetence of the host. Futo et al. (2016) reported that *Tribolium castaneum* larvae with less microbiota load showed a decrease in survival rate upon immune challenge by Bt [45], which indicates that gut microbiota is essential for immune priming. 

Another important aspect is the messengers of immune priming between hosts and microbes, which explains why different gut commensal bacteria showed a different effect on host immunity and physiology. It has been reported that peptidoglycan and uracil, which are released from intestinal commensal bacteria, can induce AMPs gene expression and ROS production to maintain the gut homeostasis [46,47,48,49]. Growing evidence revealed that messenger molecules not only involve peptidoglycan and uracil but also contain numerous bioactive compounds, such as short-chain fatty acids (SCFAs), choline metabolites, and lipids [50,51,52]. Furthermore, it has been confirmed that the axenic population of *D. melanogaster* has altered lipid metabolism and insulin signaling, but the host physiology can be restored after the administration of the gut microbial metabolite acetate [53]. A recent study found that gut microbiota may also affect the systemic immune response apart from gut immunity in Red palm weevil (RPW) *Rhynchophorus ferrugineus* Olivier larvae [44]. They might derive some metabolites, which can cross the gut epithelium and enter the host hemolymph. However, more studies are required to decipher this mechanism.

In addition to immune priming to defend microbial pathogen infection, gut commensal bacteria-mediated immune responses are also crucial for efficient arboviral acquisition in mosquitoes. Intestinal symbiotic bacterium *Serratia* Y1 has been shown to inhibit successful establishment of the *Plasmodium* through direct activation of the mosquito immune response [54], and gut microbiota could elicit a protective immune response against the *Plasmodium* transmission [55]. In contrast, *Serratia* J1, another *Serratia* strain isolated from field-caught mosquito, has no impact on *Plasmodium* development [54]. Likewise, different strains of the same bacterial species have a different effect on *Plasmodium* infections in the *Anopheles* mosquito midgut [56]. It further reminds us that the interaction between host and gut commensal bacteria is complex and may involve strain-specific outcomes according to the corresponding metabolites. 

### 2.2. Multiple Immune Reactions Help to Maintain Gut Homeostasis

**Figure 2:**
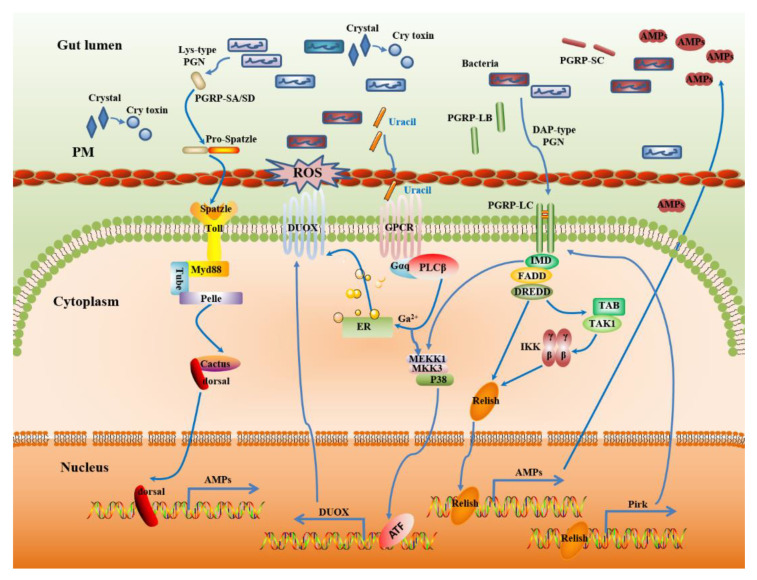
Insect gut immunity protects against infections and maintains gut microbiota homeostasis. DAP-type peptidoglycan (PGN) from intestinal bacteria is sensed by PGRP-LC, which triggers the IMD-dependent MEKK1-MKK3-p38 DUOX-expression pathway. Uracil also activates MEKK1-MKK3-p38 in a PLCβ-dependent manner; the activation of p38 enhances the transactivating function of ATF, which in turn activates the transcription of dual oxidase (DUOX). On the other hand, PLCβ-calcium signaling is responsible for the induction of DUOX enzymatic activity. Both contribute to the production of reactive oxygen species (ROS) in the gut lumen, where they control endogenous and infectious bacteria [64]. DAP-type PGN recognition by PGRP-LC also triggers the IMD pathway through the translocation of the nuclear factor-κB (NF-κB) family member Relish, which then induces increased transcription of antimicrobial peptides (AMPs) genes [23]. Besides, the IMD pathway has established a negative feedback loop to prevent overactivation. One is the members of the PGRP family gene (PGRP-LB or PGRP-SC) with amidase activity can cleave PGN and therefore blocks the activation of the IMD pathway. Another is Pirk, which interferes with the plasma membrane localization of PGRP-LC [62]. In some insect species, the Toll signaling pathway is activated with the Lys-type PGN recognition by PGRP-SA or PGRP-SD after microbial infection. This initiates a proteolytic cascade that ultimately cleaves pro-Spatzle into an active ligand for Toll, leading to the activation of the NF-κB-like transcription factors dorsal and then translocation into the nucleus to induce increased transcription of the AMP gene. Finally, these immune regulatory networks cooperatively help to maintain gut homeostasis.

The insect immune system not only protects the host against pathogen infection but also regulates the colonization of symbiotic microorganisms in the gut to maintain host homeostasis [57]. Several interesting mechanisms contribute to the proper maintenance of the microbiota by balancing the complex interaction between the host and the microbiota, which is mainly under the control of Toll and IMD pathways, and dual oxidase (DUOX) pathways, respectively (Figure 2) [23,58]. However, the functions of the Toll pathway are not consistent in different insect species, e.g., the Toll pathway is not found to be associated with the regulation of local gut immunity in *D. melanogaster* [59]. Recent reports by Abrar et al. found that the Spatzle-mediated Toll-like signaling pathway could regulate the homeostasis of gut microbiota by mediating the synthesis of AMPs in Red palm weevil, *Rhynchophorus ferrugineus* Olivier [60]. Royet et al. (2011) reported that the Toll signaling pathway could also be activated in the midgut of *P. xylostella* larvae by oral ingestion of pathogenic microbes. They also found that several essential elements for the Toll signaling pathway, including Spatzle, Toll receptor, tube, pelle, cactus, and dorsal, were expressed in *P. xylostella* midgut after the infection (Figure 2) [61]. Both Toll and IMD pathways can be activated following the detection of peptidoglycan (PGN) released from bacteria by different peptidoglycan recognition proteins (PGRPs). The family of PGRP is one of the key modulators in this process, which coordinates between the host immune response with the gut commensal bacteria. Similar to invading pathogens, gut commensal microorganisms can produce many immune-activating compounds (such as peptidoglycan) during growth and proliferation. A total of seven PGRPs were identified in *D. melanogaster* that can degrade peptidoglycan into non-immunostimulatory muropeptides. With the help of amidase activity, the peptidoglycan released from intestinal bacteria was maintained at a low basal level, so that the host can avoid the overactivation of the Toll and IMD pathway by gut microbiota (Figure 2) [62]. It has also been revealed that the low levels of peptidoglycan were limited by the PGRPs with amidase activity to transfer across the epithelial barrier and reach into hemolymph to stimulate the systematic immune response [63].

Similarly, the PGRP-LB homolog with amidase activity also acts as a negative modulator in the immunity of Red palm weevil, *Rhynchophorus ferrugineus* Olivier, in which abnormal expression alters the abundance and community structure of gut microbiota [65]. The intracellular protein Pirk can prevent PGRP-LC from recognizing extracellular peptidoglycan, thereby preventing hyperactivation of the gut immune response in flies [66]. Besides, it has been shown that peritrophic membrane (PM) integrity is related to the gut microbiota homeostasis in *A. stephensi* [67] and that PGRP-LD can help the PM to maintain structural integrity by preventing overactivation of the gut immune response, in turn limiting *P. berghei* infection. The knockdown of PGRP-LD can increase gut immunity and alters the gut microbial spatial distribution, which results in the dysbiosis of the gut microbiota. It suggests that PGRP-LD acts as a negative regulator of the immune signal pathway.

Research was also conducted to study other immune pathway regulators for maintaining gut homeostasis. Relish is an important regulator gene of the IMD pathway. Silencing the expression of Relish in the model insect *G. mellonella* results in a significant increase in the concentration of gut bacteria and decreases in the expression of AMPs [68]. Similar findings were also reported in Red palm weevil, which showed a compromised ability of pathogen clearance and increased gut bacterial load after silencing the Relish expression [69]. Moreover, a change in the gut commensal microbiota was observed after the inhibition of Caudal, a transcription repressor of NF-κB-mediated expression of AMPs [70]. The elimination of gut microbiota through antibiotics results in the downregulation of the IMD pathway and AMP gene expression [68]. Collectively, these results indicate that the IMD pathway plays a vital role in maintaining gut microbiota homeostasis.

The production of reactive oxygen species (ROS) is another inducible defense mechanism in the gut in addition to AMPs production (Figure 2) [23]. ROS are produced by the DUOX protein with an N-terminal extracellular peroxidase domain, which can convert H_2_O_2_ into HOCl in the presence of chloride, and thereby are detoxified in the presence of IRC catalase [58,71]. Unlike the gut IMD pathway, it is the uracil nucleobase, not peptidoglycan (PGN), that acts as an agonist to induce DUOX-dependent ROS production [48]. However, DUOX cannot be activated by most of the symbiotic bacteria under natural conditions, which suggest that symbiotic bacteria may block their uracil secretion pathway under natural conditions, and initiate it under specific dysregulated gut environments [48]. DUOX is also involved in the regulation of gut permeability in *Anopheles gambiae* [72]. The knockdown of DUOX increases the overall bacterial load in the oriental fruit fly *Bactrocera dorsalis;* however, the relative abundance of the bacterial symbionts *Enterobacteriaceae* is decreased in the gut [73]. 

## 3. The Host Immune System in Response to Bt Infection 

Insects can initiate humoral and cellular immune responses to reduce the damage caused by Bt infection [74,75,76,77,78,79,80]. It has been reported that Bt tolerance in the flour moth, *Ephestia kuehniella*, can be achieved by the preexposure of low-concentrated Bt endotoxins (Syngenta, North Ryde, NSW, Australia) [81]. This phenomenon is mostly denoted as immune priming, which implies that the primary exposure to pathogen activates the basic immune response result in an improved immune response upon second exposure [82,83,84]. Therefore, the mechanism of Bt toxicity does not only depend on the host receptor but is also associated with the elevated immune response of the host. It has also been reported that Bt endotoxin-tolerant *E. kuehniella* larvae can increase the lipid carrier lipophorin in the gut lumen, which inactivates Bt toxins through the aggregation of lipophorin particles to break down toxins into coagulation products [85]. A soluble toxin-binding glycoprotein is also found in the intestinal lumen of the Bt (Cry1Ac)-resistant larvae of the lepidopteran pest *Helicoverpa armigera*, which can bind to Cry1Ac and GalNAc-specific lectins and forms an insoluble aggregate [78]. An LC5 dose of Bt ssp *galleria* strain 69-6 can trigger phagocytic activity in the larvae of *Galleria melonella*, whereas an LC15 dose of Bt increases the encapsulation rate in the hemolymph during infection [86]. Similarly, both the LC15 dose and LC50 dose of Bt resulted in elevated hemolymph phenoloxidase, and lysozyme-like activity in Bt-infected *Galleria mellonella* larvae. However, the difference is that low doses of Bt can increase the humoral and cellular immune response, involve an increased encapsulation response, and enhance the phagocytic activity of hemocytes. However, a higher dose decreases cellular reactions, involving the coagulation index and activity of phenoloxidase in hemocytes [77]. The host’s immune response to Bt is likely dose dependent, as a sublethal dose of Bt damages the mid-intestinal epithelial cells, but it can be repaired by stem cell proliferation, and the enhanced immune response of the hemocytes can help to limit further infection and prevent septicemia [87]. At the LC50 dose for Bt, the situation is generally different, as the symbiotic bacteria and destroyed intestinal cells lead to dysfunctional humoral and cellular immune reactions. This indicates that Bt infection not only stimulates the local immune response in the gut but also induces the systematic immune response, where the radiation of immune pathway activation begins at the site of initial infection and radiates out, reaching the hemolymph. We found the LC50 dose of Bt infection could suppress the humoral immune response in the third instar larvae of *Plutella xylostella* [80]. Growing evidence suggests that Bt-induced immunity is a dose-dependent effect [88,89,90,91]. 

Comparatively, less information is available on the intestinal melanization response during Bt infection. It can be assumed that hemocytes can be recruited to seal perforations in the site of intestinal damage, and melanization may play a key role in this process. It has been reported that plasma phenoloxidase (PO) activity can be induced by both low and high concentrations of Bt in *G. melonella* and *E. kuehniella* larvae [77,81]. The prophenoloxidase (PPO) of insects comes from different sources and performs diverse functions, such as wound repairing, protection against pathogens, catalyzing, and detoxifying phenolics in the diet [92,93,94]. Several studies have shown that the PO may come from the hemolymph of the adult mosquito midguts [95,96]. However, other studies have shown that PPO is secreted into the foregut and can be activated by gut proteinase to detoxify phenolic present in the diet of Lepidoptera [92,93]. A recent study revealed that the PPO cascade is triggered after the infection of the Bt strain (Bt8010) in the midgut of *P. xylostella* larvae, which involves pattern recognition receptors (PRRs) and genes encoding proteases and protease inhibitors in the PPO cascade [94]. PPO can also be secreted into the hindgut to clean fecal bacteria by induced melanization of feces [92]. Similarly, the melanization response was reported in the hindgut of *Drosophila* mutant species [97]. PO-mediated melanization in the midgut might prevent symbiotic bacteria from escaping into the hemocoel through damaged midgut epithelial cells. However, despite various scientific investigations, the origin of the PO remains controversial in insects [98].

*B. thuringiensis* also synthesize another insecticidal protein (Vip) during the vegetative growth phase [99], and Vip3A, Cry1, and Cry2 genes have pyramided in cotton and maize to control lepidopteran insects [100]. The resistance to Vip3A has been selected in several lepidopteran species under laboratory conditions [101,102], while little is known of the biochemical mechanisms of resistance to Vip3A. Studies have shown this toxin does not share binding sites with Cry1 or Cry2 toxins [103,104], and in a laboratory-selected population of *Heliothis virescens*, resistance to Vip3A was shown to confer little cross-resistance to Cry1Ab and no cross-resistance to Cry1Ac [101]. The genome-wide analysis showed that most of the immune response genes, including AMPs, were upregulated, and genes involved in the metabolism and digestion process were downregulated in *Spodoptera exigua* larvae in response to Vip3 insecticidal challenge [105,106]. Similar to Bt and Bt Cry toxins, Vip3A toxin also triggers the PPO cascade and upregulates most of the genes involved in the midgut melanization process of *S. litura* and *S. exigua* [74,105]. Vip proteins also have a dose-dependent effect on the host. An increasing concentration increases the number of upregulated genes involved in the immune system and hormone modulation, and the downregulated genes involved in peritrophic membrane stability and the digestion process [106]. The genome-wide microarray analysis of Vip3Aa toxin-treated beet armyworm, *Spodoptera exigua*, showed that the upregulated enriched genes are involved in innate immune response, such as AMPs and *repat* genes [105]. This information helps to understand the host insect immune response after Bt Vip protein toxin challenge.

It is well known that the interaction between Cry toxin and toxin receptors from the host midgut brush border membrane vesicles (BBMVs) is the initial step in the insecticidal activity of the Cry protein toxins [107,108,109,110]. Several researchers also showed the interaction between midgut immune-related proteins and Cry toxin [111,112,113]. There is evidence showing that immune-related protein like Dorsal and peroxidase C in the midgut juice of *P. xylostella* and *S. exigua* can bind to the Cry1Ab1 protein toxin [111]. The protein Dorsal plays a significant role in the insect immune system, especially in the Toll pathway; therefore, a possible insecticidal mechanism of Bt Cry1Ab1 mediated by the midgut immune-related protein can be proposed. 

Similarly, it has been reported that C-type lectin-20 (CTL-20) in *Aesdes aegyptii* has the potential to bind to both toxin receptors and Cry toxins to affect the interactions between Cry toxins and toxin receptors to reduce Cry toxicity in *Aedes aegypti* [112]. Similarly, another study on immune-related peptidoglycan recognition protein (PGRP) gene expression and PO activities in Cry1Ac-susceptible and -resistant *P. xylostella* found that the resistant strain of *P. xylostella* had higher PO activity compared with the susceptible strain [114]. Moreover, among three different *P. xylostella* strains, a Cry1Ac-susceptible, a Cry1Ac–resistant strain, and a field strain, both PGRP1 (belong to PGRP-SA family) and PGRP3 (PGRP-LF) showed higher expression levels in the gut of susceptible strains compared to the resistant strain and field strains, and PGRP2 (PGRP-LB) showed the highest expression levels in the gut of resistant strains [114]. It has been found that Cry1Ah toxins can bind directly to the PPO proteins in *Ostrinia furnacalis* [115]. The interaction between Cry protein toxins and the host midgut immune-related proteins requires further investigation as the study progresses. 

The above studies have greatly enriched our knowledge of the host immune response after Bt or Bt toxin infection, and it is now confirmed that Bt or Bt toxin protein can affect the host’s immune system in a dose-dependent manner. It is known that the insect gut harbors a diverse indigenous microbiota, and the host immune system plays an important role in maintaining the gut homeostasis [42]. In the next section, we discuss the intestinal microbiota and its functions when the immune system of the insect host has been compromised by Bt or Bt toxins.

## 4. The Interaction between Bt and Host Gut Microbiota

### 4.1. B. thuringiensis Infection Altered Host Insect Gut Microbiota

In general, insects maintain a balanced local intestinal microbial community that plays a vital role in their host, including host development, nutrition, and tolerance against pathogens [116,117]. It has been shown that gut microbiota is also associated with the resistance against Bt SV2 in mosquito and Bt HD-1 in Indian meal moth, *Plodia interpunctella* (Hübner) [118,119]. The diversity and richness of gut microbiota are changed by pathogen infection. However, relatively few studies have been published on the effects of Bt or Bt toxin infection on host gut microbiota. An investigation into mosquito larvae exposed to time increasing doses of Bt showed that the lowest diversity of gut microbiota comes from the most tolerant mosquito larvae [88]. Interestingly, the same study also found that the most tolerant larvae had the highest inter-individual difference. Similarly, *B. thuringiensis* infection significantly reduced the diversity and abundance of the gut microbiota in the Bt-resistant line of *G. mellonella* [120]. However, honeybees feeding on transgenic Cry1Ah maize pollen did not result in significant changes in the gut microbiota community composition under laboratory conditions [121]. Intestinal epithelial cells act as a barrier to separate the microbiota of midgut and hemocoel, and the microbial composition differs between these two tissues under normal conditions. However, the bacterial profile between the gut and hemocoel has been reported to be similar following treatment of *Spodoptera littoralis* larvae with an LC50 dose of Bt Cry1Ca toxin [122]. This indicates that gut bacteria cross the intestinal barrier to hemolymph as a result of the Bt toxin infection and then reproduce in the insect hemolymph. However, further research is needed to reveal the action mechanism of Bt on host gut microbiota.

### 4.2. The Function of Gut Microbiota in Response to Bt Infection

The effect of insect gut microbiota in Bt toxicity has long been controversial. In 2006, Broderick et al. reported that midgut microbiota is required for Bt subspecies *kurstaki* insecticidal activity in the larvae of the gypsy moth, *Lymantria dispar*. They also found that Bt *kurstaki* was unable to multiply in insect hemolymph in vitro, indicating that intestinal bacteria cause septicemia and contribute to Bt toxicity, but without Bt, intestinal bacteria cannot induce death [21]. However, several studies showed contrasting results. In 2009, Johnston reported that intestinal bacteria were not responsible for Bt HD73 strain toxicity in the tobacco hornworm, *Manduca Sexta* [22]. Interestingly, Bt HD73 Cry^−^ cells can grow rapidly in plasma after intra-hemocoelic inoculation in many species. The same year, another study also confirmed that midgut microbiota is not required for the pathogenicity of Bt HD-73 and Bt HD-1 strains in the larvae of *P. xylostella* [123]. Work on the same insect host found that inoculation with one isolated gut bacteria *Enterobacter* sp. Mn2 has a different effect on Bt HD-1 and Bt HD-73 strain pathogenicity [123]. The contrasting results of different Bt strain pathogenicity after inoculation with the same gut bacteria to host insects indicate that we need to have an in-depth knowledge of different Bt strains before designing the experiment. From such studies, it is clear we need to pay attention to the host insect gut bacterial community, whether different diets and environments cause different gut bacteria communities between species or diverse populations within one species, and how it influences the interaction between Bt and host gut microbiota.

The interaction between Bt and gut microbiota can be competitive. *B. thuringiensis* can produce bacteriocin to inhibit the growth of gut bacteria [124]; on the other hand, insect gut microbiota can inhibit Bt multiplication, growth, and alteration of its toxins [118,125,126]: This is a kind of competition relationship. Conversely, Bt and host gut microbiota also show beneficial interactions to a certain extent; for example, some intestinal bacteria species can produce proteases that help solubilize Bt protoxins to their active form [127]. Furthermore, *B. thuringiensis* infection can promote translocation of gut-opportunistic pathogenic bacteria to hemocoel, which relies on gut epithelial damage caused by Bt toxins or some other factors, and then rapidly reproduce in the hemocoel and participate in host septicemia, finally leading to the death of the host [20,128]. One study showed that hemolymph microbiota are changed dramatically and the change is dominated by *Serratia* and *Clostridium* species upon Bt infection in *Spodoptera littoralis* larvae, which switch from asymptomatic gut symbionts to hemocoelic pathogens [122]. This translocation phenomenon agrees with the hypothesis discussed earlier in Section 4.1 of the present review.

Many gut symbiotic bacteria have been isolated and characterized; some of them showed a beneficial effect on the host and can be called probiotic. Such bacteria are widely used as animal feed additives in food production [129]. *Enterococcus mundtii* bacteria isolated from the feces of *Ephestia kuehniella* have the function of protecting the flour beetle, *Tribolium castaneum*, against Bt infection [130]. The surface properties test showed that this isolate has intense levels of auto-aggregation, which is related to the formation of colonies in the host insect’s gut [131]. Moreover, bacteria cell wall compounds, such as lipopolysaccharide (LPS) and peptidoglycan (PGN), have been well studied, and can stimulate the host immune response [132,133]. However, *Tribolium castaneum* larvae exposed to the corresponding supernatant can also increase the resistance to Bt infection [130]. This perhaps suggests that the protective function of probiotic bacteria is based on the secreted proteins or some small peptides, which may act directly against Bt infection or through the triggering of immune priming. 

Similarly, another *E. mundtii* strain isolated from *S. littoralis* also showed a protective function for the host insect, which can directly inhibit competitors and suppress pathogens’ growth through its antimicrobial activity [134]. Previous studies confirmed that *E. mundtii* cells accumulate on the surface of the intestinal epithelium and form a biofilm-like structure, which helps to keep its predominant colonization status in the host insect’s gut [135]. Additionally, after removing the dominant bacteria from the gut, this resulted in increased susceptibility of the spruce budworm larvae to Bt infection [20]. Although growing evidence confirmed that intestinal bacteria play important roles in the host defense response [136,137], the molecular function mechanism requires more research. It has been reported that *E. mundtii* SL can secrete a kind of bacteriocin, which strongly inhibits some of the competing organisms and can impair pathogen colonization in vivo [134]. Through this, we can speculate that Bt may also shown inhibited growth and has limited activity in the insect gut lumen, and the battle between Bt and probiotic *E. mundtii* may depend on the dose effect. It is also noteworthy that the secreted bacteriocin showed a selective antibacterial activity and has no influence on other intestinal bacteria, and as a result, the gut microbiota can develop normally. There is another study report that normal gut microbiota mediated pathogen clearance from the gut lumen [138]; this suggests that the gut microbiota can act as another form of protection response in organisms, or at least an important complement to host gut immune protection.

Insect gut microbiota also play an important role in Bt infection indirectly through intestinal epithelium cell regeneration. The Lepidoptera larvae intestinal epithelium mainly includes four kinds of cell types: Columnar cells, goblet cells, enteroendocrine cells, and intestinal stem cells. Each cell type has a specific role and helps maintain normal gut functions. The intestinal stem cell is the only type capable of division, which mediates epithelial renewal and the healing response [139]. *B. thuringiensis* infection can disrupt the gut epithelial cells by producing toxins, and insects mount a series of defensive responses, which involves melanization, AMP-mediated antimicrobial activity, and gut stem cell proliferation and differentiation in response to gut damage [18,94]. It has been shown that REPAT and MAPK p38 signaling pathways may be involved in the regulation of the gut defensive response to Bt toxins [18,140]. The REPAT gene was also predicted to be associated with a regenerative response in Bt-resistant insect species and showed constitutively increased expression in a Bt-resistant *S. exigua* [141]. Moreover, it showed that Cry1Ac resistance is related to an enhanced midgut healing response in the tobacco budworm [142,143]. These results suggest that intestinal stem cell activity is associated with Bt resistance because bacteria cannot get through the healthy midgut epithelial cells. This allows the host to quickly repair the damage, resulting in a limited number of invaders into the hemocoel.

It has been reported that indigenous gut microbiota can modulate the activity of intestinal stem cells in *Drosophila*, which correlates with the activated JAK/STAT pathway and epidermal growth factor receptor (EGFR) pathways [33,144]. Both the rate of epithelium renewal and the number of dividing intestinal stem cells were reduced after removing all the intestinal bacteria. The abundance of gut microbiota can also be increased after host immune suppression [145]. Interestingly, the number of mitotic intestinal stem cells is increased after blocking the *Drosophila* IMD pathway, which caused an abnormal intestinal microbiota, and many genes related to stem cell proliferation and differentiation were also upregulated by the induced gut microbiota [144]. It indicated that the intestinal stem cell proliferation could be stimulated by increased gut microbiota. In summary, these results demonstrate that insect gut microbiota can affect Bt resistance by mediated intestinal stem cell activity.

## 5. Conclusions

*B. thuringiensis* infection induces a variety of host immune responses, and interferes with the gut microbiota of the host. The resulting dysbiosis, in turn, stimulates both the expression of AMPs and the production of ROS by different ligand molecules. DUOX also plays a key role in regulating the gut microbial homeostasis. The interaction between the host immune system and gut symbionts is more cooperative rather than antagonistic. However, *B. thuringiensis* or other pathogenic infections can cause dysregulated gut environments in insects, which makes it possible to convert some symbiotic bacteria into pathobiont, known as an opportunistic pathogen. The above interaction relationship has an important effect on Bt pathogenicity or toxicity. Most studies have focused on the interaction between Bt and the host immune system or the interaction between Bt and microbiota, which significantly expands our knowledge about the dynamic Bt infection process. However, a few important aspects are still unanswered and need to be explored, i.e., (i) How does Bt trigger the immune signaling pathway? (ii) Do the membranes of the intestinal lumen or intestinal epithelial cells have any toxin recognition receptors attached to the Toll and IMD pathways? (iii) Why do different intestinal bacteria have a different effect on the host during Bt infection? and (iv) which bacterial metabolite plays a significant role in Bt toxicity and host immunocompetence?
